# The effect of COVID-19 pandemic on healthcare seeking in an urban informal settlement in Nairobi and a rural setting in western Kenya

**DOI:** 10.1371/journal.pgph.0002968

**Published:** 2024-04-17

**Authors:** George O. Agogo, Patrick K. Munywoki, Allan Audi, Joshua Auko, George Aol, Clifford Oduor, Samuel Kiplangat, Alice Ouma, Terry Komo, Amy Herman-Roloff, Peninah Munyua, Godfrey Bigogo

**Affiliations:** 1 Division of Global Health Protection, U.S. Centers for Disease Control and Prevention (CDC), Nairobi, Kenya; 2 Center for Global Health Research, Kenya Medical Research Institute (KEMRI), Kisumu, Kenya; University of Embu, KENYA

## Abstract

The COVID-19 pandemic caused widespread changes and disruptions to healthcare seeking behavior. There are limited studies on the effect of the COVID-19 pandemic on healthcare seeking patterns in low-and middle-income countries (LMICs), especially in settings with inequitable access to healthcare in rural and urban informal settlements. We investigated the effect of the COVID-19 pandemic on reported healthcare seeking at health facilities and chemists using morbidity data from participants in an ongoing population-based infectious disease surveillance platform in Asembo in Siaya County, a rural setting in western Kenya and Kibera, an urban informal settlement in Nairobi County. We described healthcare seeking patterns before (from 1^st^ January 2016 to 12^th^ March 2020) and during the pandemic (from 13^th^ March 2020 to 31^st^ August 2022) by gender and age for any reported illness and select clinical syndromes using frequencies and percentages. We used a generalized estimating equation with an exchangeable correlation structure to assess the effect of the pandemic on healthcare seeking adjusting for gender and age. Overall, there was a 19% (adjusted odds ratio, aOR: 0.81; 95% Confidence Interval, CI: 0.79–0.83) decline in odds of seeking healthcare at health facilities for any illness in Asembo during the pandemic, and a 30% (aOR: 0.70; 95% CI: 0.67–0.73) decline in Kibera. Similarly, there was a decline in seeking healthcare by clinical syndromes, e.g., for ARI, aOR: 0.76; 95% CI:0.73–0.79 in Asembo, and aOR: 0.68; 95% CI:0.64–0.72 in Kibera. The pandemic resulted in increased healthcare seeking at chemists (aOR: 1.23; 95% CI: 1.20–1.27 in Asembo, and aOR: 1.40; 95% CI: 1.35–1.46 in Kibera). This study highlights interruptions to healthcare seeking in resource-limited settings due to the COVID-19 pandemic. The pandemic resulted in a substantial decline in seeking care at health facilities, and an increase of the same at chemists.

## Introduction

Severe acute respiratory syndrome coronavirus 2 (SARS-CoV-2), the virus that causes Coronavirus disease (COVID-19), was first detected in Wuhan, Hubei province in China [1]. COVID-19 was declared a pandemic by the World Health Organization on 11 March 2020. SARS-CoV-2 spreads from an infected person via respiratory droplets [2]. The first COVID-19 case was detected in Kenya on 12 March 2020. Due to the fast spread of SARS-CoV-2, several global health agencies recommended non-pharmaceutical interventions to mitigate the spread in the early phase of the pandemic [1]. The Kenya government implemented a series of public health response measures, such as wearing of masks, practicing hand hygiene, social distancing, international travel bans, and cessation of movements in and out of areas that exhibited high rates of infections [3]. The Kenya government further expanded bed capacity, Intensive care units (ICU), and additional isolation sites to avert pressure on existing healthcare systems [3]. One year into the pandemic, COVID-19 vaccines were available globally. Kenya started COVID-19 vaccination campaigns in March 2021 and had attained 37.0% vaccination coverage for adults (aged ≥ 18 years) by December 2022 [3,4].

The COVID-19 pandemic caused widespread disruptions in healthcare seeking globally, negatively impacting healthcare utilization [5–7]. The pandemic instilled fear of getting infected in health facility settings, resulting in people opting for alternative approaches such as traditional therapies and self-treatment [8,9]. In Kenya, some health facilities were closed or partially closed and there was substantial infection among healthcare workers during the pandemic, thus offering limited healthcare services. Consequently, there have been reports of substantial reduction in healthcare utilization during the pandemic; for instance, observed reduction in healthcare activities [10], decline in emergency department (ED) visits [2], lack of, or delay in healthcare seeking among people with COVID-19-like symptoms such as shortness of breath, chest pain and hypoxia [11], and increase in utilization of telehealth services [12] in high resource settings. In similar settings, there was a reported increase in ED visits with respiratory and infection problems but a reduction in ED visits related to other non-respiratory conditions [2,13]. Fear and avoidance of infection were reported as a major hindrance to prenatal care among pregnant women in Kenya [14]. Delay or failure to seek medical care might exacerbate health problems, such as chronic diseases, leading to increased mortality [5].

Currently, there are limited studies on the effect of the COVID-19 pandemic on healthcare seeking for different clinical syndromes in low-and middle-income countries (LMICs), especially in rural and informal urban settlements settings.

In this study, we investigated the effect of the COVID-19 pandemic on healthcare seeking among individuals reporting illnesses during household visits as part of a population-based infectious disease surveillance (PBIDS) system in two diverse sites in Kenya: Asembo, Siaya County, a rural setting in western Kenya, and Kibera, an urban informal settlement in Nairobi. We characterized healthcare seeking patterns in these populations before and during the pandemic stratified by gender and age.

## Materials and methods

### Study sites

The Kenya Medical Research Institute (KEMRI) in partnership with the U.S. Centers for Disease Control and Prevention (CDC)-Kenya has been running the Population-Based Infectious Disease Surveillance (PBIDS) platform in two sites since 2006: Asembo, Siaya County, in western Kenya, and in Kibera, a densely populated urban informal settlement in Nairobi County [15]. The Asembo area is malaria-endemic, sparsely populated, and characterized by high poverty levels and high prevalence of human immunodeficiency virus (HIV, 15.3% as of 2018 [16]). The Kibera area is characterized by limited access to both healthcare and clean water [17].

### Longitudinal household-based surveillance

The PBIDS platform monitors the health of participants in the two sites and has been described in detail elsewhere [18]. In Asembo, participants reside within five kilometers of St. Elizabeth Lwak Mission Hospital, whereas in Kibera, participants reside within a one-kilometer radius of Tabitha Medical Clinic. St. Elizabeth Lwak Mission Hospital and Tabitha Medical Clinic are hereafter, referred to as surveillance health facilities. To be eligible for enrollment, persons must have resided in these study areas for at least four consecutive months or be a child born to a woman enrolled in PBIDS. Noteworthy, PBIDS participants receive free medical care at the two surveillance health facilities. For the household surveillance, trained field workers visit enrolled households and ask standardized questions about recent acute illnesses characterized by reported symptoms associated with acute respiratory, febrile, or diarrheal illnesses, experienced in the two weeks preceding the interview for each household member. For those reporting any illness, the additional enquiry is done on healthcare seeking behavior including where medical care was sought including the centrally located surveillance-supported health facilities that provide free care to participants (i.e., St. Elizabeth Lwak Mission Hospital in Asembo or Tabitha Medical Clinic in Kibera), at a non-surveillance health facility, or other sources outside of the home such as at a chemist and traditional doctors. Demographic characteristics, education, status and socio-economic data are also collected. Historically, household visits were conducted every six months since January 2016 and increased to every four months starting in September 2021. Despite changes in the frequency of household visits, the data collection tools remained the same. Noteworthy, there were no data collected between April and September 2020 due to the moratorium on field activities by the Ministry of Health to reduce SARS-CoV-2 infection in both surveillance areas. For this study, we used PBIDS data that were collected between 1^st^ January 2016 and 31^st^ August 2022, and were accessed between 1^st^ July 2022 and 30^th^ September 2023.

### Healthcare seeking and clinical syndromes definition

We defined healthcare seeking as the percent of participants with any illness or select clinical syndromes in each household visit reporting having sought healthcare from (i) surveillance health facilities, (ii) non-surveillance health facilities, or (iii) a chemist for each site. We also combined health seeking from surveillance and non-surveillance health facilities to generate healthcare seeking from any health facility. The select clinical syndromes explored were acute febrile illness (AFI), acute respiratory illness (ARI), and severe acute respiratory illness (SARI) as defined in Table 1.

**Table 1:** Case definition of the various clinical syndromes.

Case	Definitions
Acute febrile illness (AFI)** **	Participant with temperature of > = 38.0° at the time of interview or reported history of fever in the two weeks preceding the interview.
Acute respiratory illness (ARI)** **	Participant reporting cough, difficulty breathing, sore throat or chest pain when breathing in the two weeks preceding the interview.
Severe acute respiratory illness (SARI)** **	***For children < 5 years of age*:** Cough or difficulty breathing with acute onset (<2 weeks) and at least one of the following: reported fever or measured fever (≥ 38.0 C), chest indrawing, stridor in a calm child, oxygen saturation <90%, unable to breastfeed or drink, vomits everything, convulsions, lethargy, unconsciousness, admitted or referred for admission. ***For adults and children ≥ 5 years of age*** Cough or difficulty in breathing or chest pain with acute onset (<2 weeks) and at least one of the following: fever ≥ 38.0°C or reported fever, oxygen Saturation < 90%, admitted or referred for admission

### Statistical analysis

We used the PBIDS household surveillance data collected from 1^st^ January 2016 to 31^st^ August 2022 in Asembo and 1^st^ May 2016 to 31^st^ August 2022 in Kibera surveillance sites. In Kibera, data collection started in May 2016 because the enrolment of participants was still ongoing in early 2016. The prepandemic period covered 1^st^ January 2016 to 12^th^ March 2020, and the pandemic period covered 13^th^ March 2020 to 31^st^ August 2022. We assessed the effect of the COVID-19 pandemic on healthcare seeking patterns for any reported illness and select clinical syndromes (AFI, ARI and SARI), adjusting for gender and age. First, we described reported healthcare seeking before and during the COVID-19 pandemic at any health facility (surveillance and non-surveillance), surveillance facilities only, and at chemists using frequencies and percentages, stratified by gender and age. Second, we assessed factors associated with healthcare seeking using a Generalized estimating equation (GEE) with an exchangeable working correlation to account for the within-person clustering, and quantified the marginal effects with the odds ratios estimates [19]. The GEE approach uses empirical covariance estimator for the fixed effects. The empirical estimators are robust against the choice of working covariance structure. The model parameters were estimated by residual pseudo-likelihood using Newton-Raphson optimization with ridging. In assessing the effect of the COVID-19 pandemic on healthcare seeking, we adjusted the multivariable GEE model for gender and age categories to obtain adjusted odds ratio (aOR) estimates to quantify the pandemic effect (relative to the prepandemic). We conducted site-specific as well as combined regression analyses for healthcare seeking due to any reported illness and select clinical syndromes. In the combined analysis, we combined the data from both sites and further adjusted for the site effect in the model.

We fitted the GEE model using the GLIMMIX procedure in SAS software version 9.4 (SAS Institute Inc, Cary, NC). The level of significance was defined as α ≤ 0.05.

### Ethical considerations

The PBIDS protocol was reviewed and approved by the Institutional review boards of KEMRI (SSC #1899 and #2761) and CDC (#4566 and #6775). Written informed consent was obtained from the head of household at the time of enrollment into PBIDS in Asembo and Kibera.

## Results

### Baseline data

**Fig 1:**
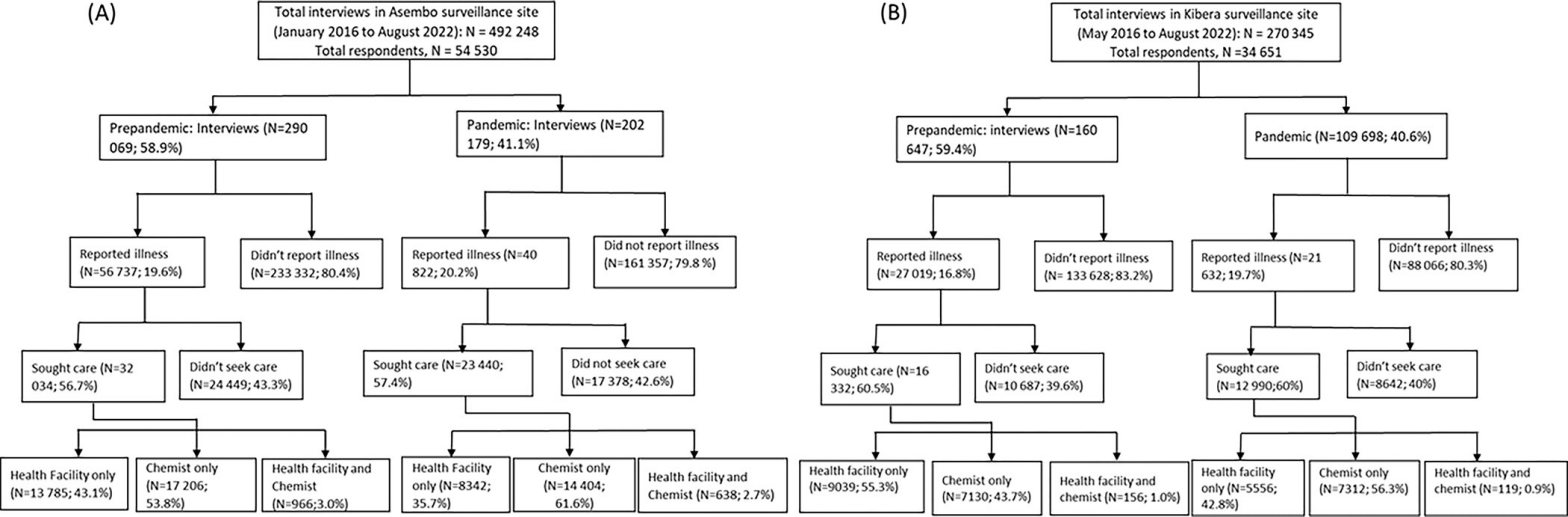
Flowchart showing the frequency distributions of interviews, reported illness, and reported healthcare seeking at health facility and chemist visits in Asembo (A) and Kibera (B).

We used healthcare seeking data from 54,530 participants (from 492,248 interviews) in Asembo collected from 1^st^ January 2016 to 31^st^ August 2022 and 34,651 participants (from 270,345 interviews) in Kibera from 1^st^ May 2016 to 31^st^ August 2022. The distribution of the interviews, reported illnesses, and report of healthcare seeking with any illness for Asembo and Kibera, respectively, are shown in Fig 1.

### Reported healthcare seeking in health facilities and at chemists before and during the COVID-19 pandemic in Asembo and Kibera

**Fig 2:**
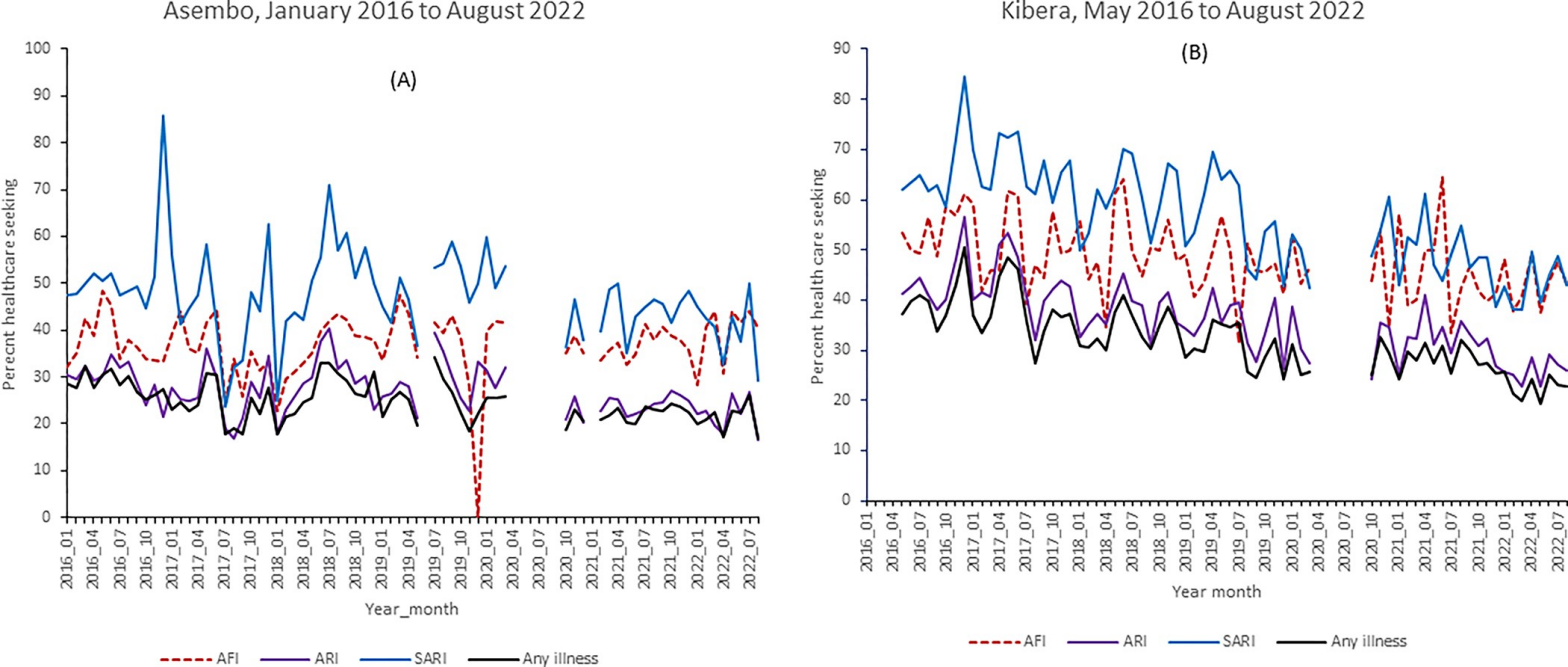
Monthly-delimited percent reporting healthcare seeking at a health facility among those with any illness, AFI, ARI and SARI in Asembo, Siaya County (A) and Kibera, Nairobi County (B). A health facility care seeking is defined as those who reported seeking care at the surveillance and non-surveillance health facilities.

Among all interviews in Asembo, there was an increase in percent reported illnesses during the pandemic compared to the prepandemic period (20.2% vs. 19.6%, P-value < 0.0001). In contrast, the percent seeking healthcare at any health facility with any illness decreased during the pandemic compared to the prepandemic period (35.7% vs. 43.1%, P-value < 0.0001). Participants seeking healthcare at chemists increased from 53.8% prepandemic to 61.6% in the pandemic period in Asembo (Fig 1A, P-value < 0.0001). In Kibera, percent reported illnesses increased from 16.8% to 19.7% (P-value < 0.0001), percent reported healthcare seeking at any health facility decreased from 55.3% to 42.3% (P-value < 0.0001) and percent healthcare seeking at chemists increased from 43.7% to 56.3% during the pandemic (Fig 1B, P-value < 0.0001). The temporal patterns showing the monthly variability in the percent reported healthcare seeking for any illness and select clinical syndromes is shown in Fig 2.

For the select clinical syndromes in Asembo, we observed a decline in the percent seeking healthcare at any health facility during the pandemic with ARI (23.6% vs 29.4%, P-value < 0.0001) and SARI (43.8% vs 49.7%, P-value < 0.0001, Table 2) relative to the prepandemic period, but no change for those with AFI (38.2% vs 38.2%, P-value = 0.97). In contrast, participants reporting having sought healthcare at the surveillance health facility in Asembo with AFI, and SARI was higher during the pandemic (15.2% vs 12.9% for AFI, P-value < 0.0001 and 18.8% vs 17.8% for SARI, P-value = 0.073, Table 3), but was lower for those with ARI (9.6% vs 10.3%, P-value = 0.035) compared to the prepandemic period.

**Table 2:** Reported healthcare seeking at a health facility or chemist with any illness or select clinical syndromes before and during COVID-19 pandemic in Asembo, Siaya County and Kibera, Nairobi County, Kenya.

Syndromes & Characteristics	Asembo						Kibera					
	*Health facility*, *n (%)*^*a*^			*Chemist*, *n (%)*			*Health facility*, *n (%)*			*Chemist*, *n (%)*		
	Prepandemic	Pandemic	P-value	Prepandemic	Pandemic	P-value	Prepandemic	Pandemic	P-value	Prepandemic	Pandemic	P-value
***Any illness***	* *	* *	* *	* *	* *	* *	* *	* *	* *	* *	* *	
Overall	14 828(26.1)	9036 (22.1)	< 0.0001	18172(32.2)	15042(37.0)	< 0.0001	9202 (34.1)	5678(26.3)	< 0.0001	7286 (27.0)	7431(34.4)	< 0.0001
Gender												
• Female	8631 (25.7)	5210 (21.9)	< 0.0001	10 542(31.6)	8590 (36.3)	< 0.0001	5369 (35.3)	3350(27.5)	< 0.0001	3915 (25.8)	4008(32.9)	< 0.0001
• Male	6197 (26.7)	3826 (22.4)	< 0.0001	7630(33.1)	6452 (38.1)	< 0.0001	3833 (32.4)	2328(24.7)	< 0.0001	3371 (28.6)	3423(36.3)	< 0.0001
Age category (years)												
• < 5	3557 (32.4)	2019 (28.1)	< 0.0001	3214(29.4)	2703 (37.8)	< 0.0001	3044 (42.9)	1665(32.5)	< 0.0001	1636 (23.1)	1707(33.4)	< 0.0001
• 5–17	5189 (29.3)	3156 (24.8)	< 0.0001	5833(33.1)	5100 (40.4)	< 0.0001	2217 (30.7)	1364(23.6)	< 0.0001	1906 (26.4)	1972(34.1)	< 0.0001
• 18–29	1734 (28.0)	1103 (24.0)	< 0.0001	2025(33.0)	1641 (35.9)	0.001	1450 (30.5)	954(24.0)	< 0.0001	1330 (28.1)	1363(34.3)	< 0.0001
• 30–39	1012 (23.0)	641 (19.3)	< 0.0001	1543(35.5)	1294 (39.2)	0.001	1021 (29.2)	698(24.2)	< 0.0001	1104 (31.5)	1054(36.6)	< 0.0001
• 40–49	754 (21.6)	506 (17.4)	< 0.0001	1285(37.0)	1140 (39.5)	0.039	803 (32.2)	549(25.6)	< 0.0001	769 (30.9)	785 (36.6)	< 0.0001
• 50–59	850 (21.6)	554 (19.4)	0.024	1327(34.0)	1036 (36.4)	0.040	469 (33.4)	288(24.3)	< 0.0001	404 (28.8)	406 (34.3)	0.003
• 60+	1732 (17.3)	1057 (14.6)	< 0.0001	2945(29.5)	2128 (29.5)	0.999	198 (35.6)	160(30.1)	0.057	137 (24.6)	144 (27.2)	0.333
***AFI***												
Overall	4966 (38.2)	3375 (38.2)	0.970	4579(35.3)	3823 (43.3)	< 0.0001	1667 (49.8)	937 (43.4)	< 0.0001	997 (29.8)	780 (36.1)	< 0.0001
Gender												
• Female	2809 (39.3)	1871 (39.3)	0.997	2424(34.0)	2002 (42.1)	< 0.0001	997 (52.6)	512 (44.4)	< 0.0001	524 (27.6)	393 (34.1)	0.0002
• Male	2157 (36.8)	1504 (36.9)	0.913	2155(36.8)	1821 (44.7)	< 0.0001	670 (46.1)	425 (42.2)	0.051	473 (32.6)	387 (38.4)	0.003
Age category (years)												
• < 5	1170 (39.2)	775 (39.7)	0.757	912 (30.6)	769 (39.4)	< 0.0001	373 (49.7)	241 (44.9)	0.090	206 (27.4)	189 (35.2)	0.003
• 5–17	2188 (37.9)	1509 (36.7)	0.244	2175(37.7)	1900 (46.2)	< 0.0001	473 (50.2)	259 (43.8)	0.014	297 (31.6)	220 (37.2)	0.025
• 18–29	582 (43.5)	426 (45.9)	0.263	452 (33.9)	363 (39.1)	0.011	309 (52.2)	170 (45.5)	0.041	164 (27.7)	133 (35.6)	0.010
• 30–39	308 (38.9)	200 (38.1)	0.772	298 (37.7)	241 (45.9)	0.003	227 (46.0)	109 (39.4)	0.076	160 (32.4)	111 (40.1)	0.032
• 40–49	205 (37.4)	142 (36.0)	0.647	217 (39.7)	184 (46.6)	0.034	150 (47.3)	93 (43.1)	0.332	100 (31.6)	71 (32.9)	0.748
• 50–59	206 (37.3)	152 (41.4)	0.212	182 (33.0)	147 (40.1)	0.028	97 (51.6)	45 (37.8)	0.018	60 (31.9)	45 (37.8)	0.288
• 60+	307 (30.4)	171 (31.0)	0.784	343 (33.9)	219 (39.8)	0.022	38 (59.4)	20 (43.5)	0.100	10 (15.6)	11 (23.9)	0.275
**ARI**												
Overall	7236 (29.4)	3921 (23.6)	< 0.0001	8428(34.4)	6655 (40.3)	< 0.0001	5854 (38.9)	3365(29.8)	< 0.0001	4212 (28.0)	4076(36.2)	< 0.0001
Gender												
• Female	4239 (29.4)	2294 (24.0)	< 0.0001	4865 (33.9)	3749 (39.5)	< 0.0001	3368 (40.0)	2007(31.2)	< 0.0001	2246 (26.7)	2248(35.0)	< 0.0001
• Male	2997 (29.3)	1627 (23.1)	< 0.0001	3563 (35.0)	2906 (41.4)	< 0.0001	2486 (37.5)	1358(28.1)	< 0.0001	1966 (29.7)	1828(37.8)	< 0.0001
Age category (years)												
• <5	2170 (38.3)	1105 (31.8)	< 0.0001	1850 (32.7)	1480 (42.8)	< 0.0001	2395 (51.1)	1206(38.8)	< 0.0001	1145 (24.4)	1094(35.2)	< 0.0001
• 5–17	2406 (30.6)	1225 (23.8)	< 0.0001	2643 (33.8)	2143 (41.9)	< 0.0001	1370 (34.8)	823 (26.7)	< 0.0001	1102 (28.0)	1136(37.0)	< 0.0001
• 18–29	741 (29.1)	417 (24.4)	0.001	906 (35.8)	648 (38.1)	0.127	819 (32.9)	479 (25.4)	< 0.0001	716 (28.9)	666 (35.2)	< 0.0001
• 30–39	441 (24.6)	258 (19.7)	0.001	692 (38.9)	550 (42.2)	0.068	537 (30.0)	350 (25.4)	0.004	589 (33.0)	526 (38.2)	0.002
• 40–49	320 (22.4)	224 (19.0)	0.037	566 (39.7)	522 (44.7)	0.011	425 (35.0)	287 (27.8)	0.0002	389 (32.1)	394 (38.1)	0.003
• 50–59	376 (25.1)	215 (20.5)	0.006	561 (37.7)	444 (42.3)	0.019	214 (31.9)	146 (26.4)	0.037	205 (30.5)	192 (34.9)	0.107
• 60+	782 (20.3)	477 (17.5)	0.004	1210 (31.6)	868 (32.0)	0.772	94 (35.9)	74 (32.5)	0.426	66 (25.2)	68 (29.8)	0.252
**SARI**												
Overall	4432 (49.7)	2206 (43.8)	< 0.0001	2953 (33.1)	2115 (42.0)	< 0.0001	4141 (60.7)	1992(47.1)	< 0.0001	1634 (24.0)	1463(34.6)	< 0.0001
Gender												
• Female	2438 (49.7)	1189 (43.6)	< 0.0001	1595 (32.5)	1117 (41.0)	< 0.0001	2248 (61.3)	1136(49.1)	< 0.0001	850 (23.2)	777 (33.6)	< 0.0001
• Male	1994 (49.8)	1017 (43.9)	< 0.0001	1358 (33.9)	998 (43.2)	< 0.0001	1893 (60.1)	856 (44.7)	< 0.0001	784 (24.9)	686 (35.8)	< 0.0001
Age category(years)												
• < 5	1676 (53.8)	830 (48.1)	0.0002	944 (30.3)	695 (40.4)	< 0.0001	2112 (62.9)	982 (49.1)	< 0.0001	742 (22.1)	686 (34.3)	< 0.0001
• 5–17	1657 (52.6)	772 (43.1)	< 0.0001	1035 (32.8)	789 (44.0)	< 0.0001	939 (61.7)	447 (48.0)	< 0.0001	385 (25.3)	325 (34.9)	< 0.0001
• 18–29	333 (50.6)	185 (47.3)	0.302	238 (36.2)	154 (39.4)	0.298	395 (57.2)	213 (46.4)	0.0003	170 (24.6)	151 (32.9)	0.002
• 30–39	170 (42.4)	110 (40.2)	0.561	160 (39.9)	131 (47.8)	0.042	285 (55.3)	136 (41.7)	0.0001	142 (27.6)	116 (35.6)	0.014
• 40–49	150 (41.3)	83 (32.8)	0.032	140 (38.6)	127 (50.2)	0.004	249 (58.2)	134 (43.4)	< 0.0001	105 (24.5)	115 (37.2)	0.0002
• 50–59	163 (42.0)	84 (45.4)	0.443	151 (38.9)	76 (41.1)	0.621	121 (53.3)	52 (35.9)	0.001	67 (29.5)	55 (37.9)	0.092
• 60+	283 (33.9)	142 (33.7)	0.932	285 (34.2)	143 (34.0)	0.942	40 (52.0)	28 (46.7)	0.540	23 (29.9)	15 (25.0)	0.528

AFI, Acute febrile illness; ARI, Acute Respiratory Illness; SARI, Severe Acute Respiratory Illness; ^***a***^, n (%) denotes the number (and percent) of healthcare sought among reported illnesses.

**Table 3:** Reported healthcare seeking at surveillance and non-surveillance health facilities with any or select clinical syndromes before and during COVID-19 pandemic in Asembo, Siaya County and Kibera, Nairobi County, Kenya.

	Asembo						Kibera					
Syndromes &	*Surveillance HF*, *n (%)*^*a*^			*Non-surveillance HF*, *n (%)*			*Surveillance HF*, *n (%)*			*Non-surveillance HF*, *n (%)*		
Characteristics	*Prepandemic*	*Pandemic*	P-value	*Prepandemic*	*Pandemic*	P-value	*Prepandemic*	*Pandemic*	P-value	*Prepandemic*	*Pandemic*	P-value
**Any illness**												
Overall	5030 (8.9)	3511 (8.6)	0.149	13 437 (23.7)	9002 (22.1)	<0.0001	5258 (19.5)	3255(15.1)	<0.0001	4149 (15.4)	2483 (11.5)	<0.0001
Gender												
• Female	2977 (8.9)	2116 (8.9)	0.930	7830 (23.4)	5188 (21.8)	<0.0001	3071 (20.2)	1951(16.0)	<0.0001	2414 (15.9)	1436 (11.8)	<0.0001
• Male	2053 (8.8)	1395 (8.2)	0.019	5607 (24.2)	3814 (22.4)	<0.0001	2187 (18.5)	1304(13.8)	<0.0001	1735 (14.7)	1047 (11.1)	<0.0001
Age category(years)												
• < 5	1293 (11.8)	867 (12.1)	0.572	3236 (29.5)	2014 (28.0)	0.0321	1754 (24.7)	974 (19.0)	<0.0001	1364 (19.2)	702 (13.7)	<0.0001
5–17	1584 (8.9)	1131 (8.9)	0.903	4765 (26.9)	3146 (24.7)	<0.0001	1313 (18.2)	802 (13.9)	<0.0001	947 (13.1)	577 (10.0)	<0.0001
• 18–29	568 (9.2)	421 (9.2)	0.993	1579 (25.5)	1094 (23.8)	0.050	822 (17.3)	573 (14.4)	0.0002	655 (13.8)	392 (9.9)	<0.0001
• 30–39	392 (8.9)	291 (8.7)	0.778	888 (20.2)	638 (19.2)	0.250	551 (15.7)	346 (12.0)	<0.0001	497 (14.2)	361 (12.5)	0.051
• 40–49	258 (7.4)	187 (6.5)	0.141	683 (19.6)	503 (17.3)	0.023	444 (17.8)	320 (14.9)	0.008	381 (15.3)	238 (11.1)	<0.0001
• 50–59	285 (7.3)	196 (6.9)	0.534	747 (19.0)	552 (19.3)	0.748	261 (18.6)	151 (12.7)	<0.0001	218 (15.5)	140 (11.8)	0.007
• 60+	650 (6.5)	418 (5.8)	0.056	1539 (15.4)	1055 (14.6)	0.157	113 (20.3)	89 (16.8)	0.135	87 (15.6)	73 (13.8)	0.384
**AFI**												
Overall	1674 (12.9)	1346 (15.2)	< 0.0001	4512 (34.7)	3374 (38.2)	<0.0001	868 (25.9)	511 (23.7)	0.057	844 (25.2)	444 (20.6)	< .0001
Gender												
• Female	987 (13.8)	778 (16.4)	0.0001	2549 (35.7)	1870 (39.3)	<0.0001	519 (27.4)	278 (24.1)	0.047	507 (26.7)	244 (21.2)	0.001
• Male	687 (11.7)	568 (13.9)	0.001	1963 (33.5)	1504 (36.9)	0.0004	349 (24.0)	233 (23.1)	0.597	337 (23.2)	200 (19.8)	0.047
Age category (years)												
• < 5	430 (14.4)	335 (17.1)	0.010	1063 (35.6)	775 (39.7)	0.004	175 (23.3)	136 (25.3)	0.403	207 (27.6)	107 (19.9)	0.002
• 5–17	666 (11.5)	570 (13.9)	0.001	2007 (34.7)	1508 (36.7)	0.045	250 (26.5)	141 (23.8)	0.234	236 (25.1)	125 (21.1)	0.077
• 18–29	200 (15.0)	164 (17.7)	0.084	528 (39.5)	426 (45.9)	0.002	162 (27.4)	89 (23.8)	0.218	153 (25.8)	84 (22.5)	0.234
• 30–39	117 (14.8)	83 (15.8)	0.608	277 (35.0)	200 (38.1)	0.249	113 (22.9)	57 (50.6)	0.461	123 (24.9)	57 (20.6)	0.174
• 40–49	72 (13.1)	58 (14.7)	0.497	183 (33.4)	142 (36.0)	0.415	88 (27.8)	52 (24.1)	0.342	68 (21.5)	42 (19.4)	0.574
• 50–59	71 (12.9)	60 (16.4)	0.139	184 (33.3)	152 (41.4)	0.013	57 (30.3)	20 (16.8)	0.008	42 (22.3)	25 (21.0)	0.783
• 60+	118 (11.7)	76 (13.8)	0.225	270 (26.7)	171 (31.0)	0.069	23 (35.9)	16 (34.8)	0.901	15 (23.4)	4 (8.7)	0.072
**ARI**												
Overall	2528 (10.3)	1596 (9.6)	0.035	6600 (26.8)	3906 (23.5)	<0.0001	3528 (23.4)	1990(17.6)	< .0001	2454 (16.3)	1407 (12.5)	< .0001
Gender												
• Female	1491 (10.3)	973 (10.2)	0.737	3874 (26.9)	2285 (24.0)	<0.0001	2037 (24.2)	1205(18.7)	< .0001	1399 (16.6)	823 (12.8)	< .0001
• Male	1037 (10.1)	623 (8.8)	0.004	2726 (26.7)	1621 (23.0)	<0.0001	1491 (22.5)	785 (16.2)	< .0001	1055 (15.9)	584 (12.1)	< .0001
Age category (years)												
• <5	781 (13.8)	480 (13.8)	0.977	1985 (35.0)	1100 (31.6)	0.001	1431 (30.5)	722 (23.2)	< .0001	1024 (21.9)	493 (15.9)	< .0001
• 5–17	773 (9.8)	459 (8.9)	0.088	2218 (28.2)	1223 (23.8)	<0.0001	861 (21.9)	491 (16.0)	< .0001	530 (13.5)	338 (11.0)	0.002
• 18–29	245 (9.6)	162 (9.5)	0.881	674 (26.4)	414 (24.2)	0.102	491 (19.7)	309 (16.3)	0.004	342 (13.7)	174 (9.2)	< .0001
• 30–39	172 (9.6)	127 (9.7)	0.932	389 (21.7)	256 (19.5)	0.140	311 (17.4)	182 (13.2)	0.001	240 (13.4)	172 (12.5)	0.430
• 40–49	110 (7.7)	86 (7.3)	0.714	294 (20.6)	222 (18.9)	0.283	247 (20.4)	166 (16.1)	0.009	190 (15.7)	126 (12.2)	0.018
• 50–59	129 (8.6)	75 (7.1)	0.174	330 (22.1)	215 (20.5)	0.332	127 (18.9)	82 (14.8)	0.060	93 (13.8)	66 (11.9)	0.324
• 60+	318 (8.3)	207 (7.6)	0.323	710 (18.5)	476 (17.5)	0.306	60 (22.9)	38 (16.7)	0.085	35 (13.4)	38 (16.7)	0.305
**SARI**												
Overall	1568 (17.8)	948 (18.8)	0.073	4087 (45.9)	2205 (43.7)	0.016	2578 (37.8)	1215(28.7)	< .0001	1655 (24.3)	797 (18.8)	< .0001
Gender												
• Female	860 (17.5)	521 (19.1)	0.083	2253 (45.9)	1189 (43.6)	0.056	1400 (38.2)	709 (30.6)	< .0001	892 (24.3)	441 (19.0)	< .0001
• Male	708 (17.7)	427 (18.4)	0.446	1834 (45.8)	1016 (43.9)	0.141	1178 (37.4)	506 (26.4)	< .0001	763 (24.2)	356 (18.6)	< .0001
Age category(years)												
• < 5	602 (19.31)	376 (21.8)	0.038	1544 (49.5)	829 (48.1)	0.334	1285 (38.3)	605 (30.2)	< .0001	881 (26.2)	385 (19.2)	< .0001
• 5–17	556 (17.6)	300 (16.7)	0.422	1541 (48.9)	772 (43.1)	<0.0001	613 (40.3)	277 (29.7)	< .0001	341 (22.4)	174 (18.7)	0.027
• 18–29	111 (16.9)	74 (18.9)	0.398	305 (46.4)	185 (47.3)	0.763	256 (37.1)	140 (30.5)	0.022	146 (21.1)	76 (16.6)	0.054
• 30–39	68 (17.0)	58 (21.2)	0.168	159 (39.7)	110 (40.2)	0.897	171 (33.2)	75 (23.0)	0.002	121 (23.5)	61 (18.7)	0.101
• 40–49	63 (17.4)	43 (17.0)	0.908	140 (38.6)	83 (32.8)	0.143	157 (36.7)	73 (23.6)	0.0002	98 (22.9)	63 (20.4)	0.416
• 50–59	54 (13.9)	33 (17.8)	0.221	139 (35.8)	84 (45.4)	0.028	71 (31.3)	29 (20.0)	0.017	53 (23.4)	24 (16.6)	0.115
• 60+	114 (13.7)	64 (15.2)	0.467	259 (31.0)	142 (33.7)	0.345	25 (32.5)	16 (26.7)	0.462	15 (19.5)	14 (23.3)	0.584

AFI, Acute febrile illness; ARI, Acute Respiratory Illness; SARI, Severe Acute Respiratory Illness; HF, health facility; ^***a***^, n (%) denotes the number (and percent) of healthcare sought among reported illnesses.

Relative to the prepandemic period, we observed a decline in the percentage of individuals who reported seeking healthcare at any health facility during the pandemic with AFI (43.4% vs 49.8%, P-value < 0.0001), ARI (29.8% vs 38.9%, P-value < 0.0001), and SARI (47.1% vs 60.7%, P-value < 0.0001, Table 2) in Kibera informal settlement. Similarly, there was a decline in those who reported seeking healthcare at the surveillance health facility in Kibera during the pandemic for the three clinical syndromes (23.7% vs 25.9% for AFI, P-value = 0.057; 17.6% vs 23.4% for ARI, P-value < 0.0001; and 28.7% vs 37.8% for SARI, P-value < 0.0001, Table 3).

### Comparison of reported healthcare seeking during the COVID-19 pandemic compared to prepandemic period using the multivariable GEE regression analysis

The adjusted odds ratios from the multivariable GEE regression analyses for reported healthcare seeking with any illness or with the select clinical syndromes relative to prepandemic period are presented in Table 4. Relative to the prepandemic period, Asembo participants had lower odds of reporting healthcare seeking at a health facility for any illness (aOR: 0.81; 95% CI: 0.79–0.83), ARI (aOR: 0.76; 95% CI: 0.73–0.79), and SARI (aOR: 0.78; 95% CI: 0.73–0.84) in the pandemic period. There was no statistically significant difference in the odds of reporting healthcare seeking with AFI in pandemic period relative to the prepandemic period (aOR: 0.99; 95% CI: 0.94–1.05). For Kibera, there was a decline in reported healthcare seeking at any health facility with any illness or with ARI, SARI and AFI, Table 4. From the combined analysis, the participants in Kibera were more likely to report healthcare seeking at any health facility with any illness relative to those in Asembo (aOR: 1.26; 95% CI: 1.22–1.30).

**Table 4:** Effect of COVID-19 pandemic on reported healthcare seeking at health facilities with any illness and select clinical syndromes adjusted for age and gender in Asembo, Siaya County and Kibera, Nairobi County, Kenya.

Syndromes		Asembo			Kibera			Both sites		
	Characteristic variables	aOR	95% CI		aOR	95% CI		aOR	95% CI	
Any illness	Pandemic (ref prepandemic)	0.809	0.785	0.834	0.698	0.670	0.727	0.768	0.750	0.787
	Site (Kibera vs Asembo)							1.260	1.224	1.296
	Male (vs female)	0.930	0.898	0.962	0.829	0.792	0.867	0.905	0.881	0.930
	Age category (ref < 5 years)									
	• 5–17	0.852	0.816	0.889	0.609	0.575	0.645	0.758	0.732	0.784
	• 18–29	0.798	0.754	0.845	0.585	0.547	0.626	0.700	0.670	0.731
	• 30–39	0.612	0.572	0.654	0.566	0.525	0.611	0.597	0.568	0.628
	• 40–49	0.549	0.509	0.592	0.636	0.585	0.692	0.592	0.560	0.627
	• 50–59	0.572	0.530	0.618	0.674	0.606	0.749	0.590	0.555	0.627
	• 60+	0.437	0.412	0.465	0.793	0.680	0.925	0.445	0.421	0.470
	Pandemic (ref prepandemic)	0.990	0.936	1.047	0.767	0.684	0.860	0.940	0.894	0.988
	Site (Kibera vs Asembo)							1.408	1.295	1.531
	Male (vs female)	0.898	0.847	0.952	0.823	0.736	0.922	0.883	0.812	0.961
	Age category (ref < 5 years)									
AFI	• 5–17	0.906	0.844	0.973	0.960	0.822	1.121	0.917	0.860	0.979
	• 18–29	1.196	1.079	1.326	1.008	0.846	1.201	1.143	1.046	1.250
	• 30–39	0.943	0.829	1.073	0.776	0.643	0.938	0.888	0.799	0.987
	• 40–49	0.890	0.767	1.031	0.885	0.714	1.096	0.893	0.792	1.007
	• 50–59	0.951	0.819	1.103	0.927	0.712	1.207	0.945	0.830	1.075
	• 60+	0.659	0.581	0.749	1.253	0.843	1.864	0.689	0.612	0.776
	Pandemic (vs prepandemic)	0.759	0.725	0.794	0.681	0.644	0.719	0.725	0.700	0.751
ARI	Site (Kibera vs Asembo)							1.366	1.315	1.419
	Male (vs female)	0.878	0.836	0.922	0.807	0.762	0.856	0.858	0.826	0.890
	Age category (ref < 5 years)									
	• 5–17	0.700	0.659	0.744	0.536	0.499	0.576	0.627	0.599	0.656
	• 18–29	0.657	0.604	0.715	0.465	0.426	0.507	0.555	0.523	0.590
	• 30–39	0.515	0.466	0.568	0.433	0.392	0.478	0.475	0.443	0.510
	• 40–49	0.469	0.421	0.522	0.525	0.470	0.576	0.500	0.463	0.541
	• 50–59	0.523	0.469	0.584	0.502	0.433	0.581	0.505	0.463	0.551
	• 60+	0.426	0.392	0.464	0.637	0.517	0.783	0.423	0.392	0.456
	Pandemic (vs prepandemic)	0.781	0.727	0.838	0.581	0.537	0.630	0.686	0.650	0.723
	Site (Kibera vs Asembo)							1.330	1.259	1.404
SARI	Male	0.928	0.864	0.997	0.868	0.800	0.942	0.906	0.858	0.956
	Age category (ref < 5 years)									
	• 5–17	0.894	0.823	0.971	0.952	0.861	1.053	0.921	0.864	0.981
	• 18–29	0.898	0.782	1.031	0.808	0.704	0.928	0.852	0.773	0.940
	• 30–39	0.657	0.555	0.779	0.709	0.607	0.829	0.688	0.614	0.771
	• 40–49	0.577	0.484	0.687	0.795	0.673	0.938	0.687	0.610	0.774
	• 50–59	0.675	0.562	0.811	0.657	0.525	0.823	0.666	0.577	0.767
	• 60+	0.467	0.407	0.537	0.774	0.547	1.094	0.497	0.438	0.563

AFI, Acute febrile illness; ARI, Acute Respiratory Illness; SARI, Severe Acute Respiratory Illness; aOR, adjusted odds ratio; CI, confidence interval.

The site-specific adjusted effect estimates of reported healthcare seeking at the surveillance health facilities with any illness or select clinical syndromes are presented in Table 5. In Asembo, we observed a marginal decline (aOR: 0.95; 95% CI: 0.91–1.00) in the odds of reported healthcare seeking at St. Elizabeth Lwak Mission Hospital with any illness, but an 18% (aOR: 1.18; 95% CI: 1.09–1.28) increase for those with AFI during the pandemic compared to the prepandemic period. In Kibera, there was a decline in the odds of reported healthcare seeking at Tabitha Medical Clinic with any illness or with the select clinical syndromes during the pandemic relative to the prepandemic period (aORs < 1, Table 5). For the combined analysis, the odds of reported healthcare seeking at the Tabitha Medical Clinic with any illness was twice the odds of healthcare seeking at St. Elizabeth Lwak Mission Hospital (aOR: 2.06; 95% CI: 1.98–2.14).

**Table 5:** Effect of COVID-19 pandemic on healthcare seeking at surveillance health facilities with any illness and select clinical syndromes adjusted for age and gender in Asembo, Siaya County and Kibera, Nairobi County, Kenya.

Syndromes	Characteristic variables	St. Elizabeth Lwak Hospital			Tabitha Medical Clinic			Both sites		
		aOR	95% CI		aOR	95% CI		aOR	95% CI	
Any illness	• Pandemic (vs prepandemic)	0.953	0.910	0.997	0.744	0.707	0.784	0.850	0.821	0.880
	• Site (Kibera vs Asembo)							2.058	1.978	2.141
	• Male (vs female)	0.880	0.834	0.929	0.833	0.788	0.881	0.869	0.836	0.903
	Age category (ref < 5 years)									
	• 5–17	0.750	0.701	0.802	0.678	0.632	0.728	0.717	0.683	0.753
	• 18–29	0.745	0.682	0.813	0.644	0.594	0.699	0.691	0.651	0.734
	• 30–39	0.724	0.655	0.800	0.556	0.505	0.612	0.627	0.585	0.673
	• 40–49	0.571	0.508	0.641	0.660	0.594	0.735	0.623	0.576	0.674
	• 50–59	0.562	0.499	0.633	0.664	0.580	0.760	0.598	0.547	0.653
	• 60+	0.491	0.447	0.539	0.794	0.648	0.974	0.519	0.478	0.563
	• Pandemic (vs prepandemic)	1.180	1.091	1.277	0.866	0.759	0.989	1.083	1.012	1.159
AFI	• Site (Kibera vs Asembo)							2.043	1.843	2.264
	• Male (vs female)	0.827	0.760	0.900	0.879	0.773	1.000	0.845	0.754	0.947
	Age category (ref < 5 years)									
	• 5–17	0.773	0.698	0.856	1.054	0.882	1.259	0.838	0.767	0.915
	• 18–29	0.991	0.859	1.143	1.061	0.869	1.295	1.006	0.896	1.129
	• 30–39	0.952	0.798	1.134	0.836	0.670	1.044	0.880	0.767	1.011
	• 40–49	0.875	0.712	1.076	1.080	0.846	1.378	0.943	0.807	1.101
	• 50–59	0.869	0.704	1.073	1.001	0.737	1.358	0.912	0.767	1.084
	• 60+	0.742	0.622	0.886	1.694	1.119	2.565	0.841	0.716	0.987
	• Pandemic (vs prepandemic)	0.937	0.876	1.003	0.717	0.671	0.765	0.813	0.775	0.852
ARI	• Site (Kibera vs Asembo)							2.264	2.151	2.384
	• Male (vs female)	0.858	0.797	0.924	0.820	0765	0.878	0.846	0.804	0.889
	Age category (ref < 5 years)									
	• 5–17	0.681	0.622	0.745	0.633	0.581	0.688	0.654	0.615	0.695
	• 18–29	0.654	0.577	0.742	0.562	0.508	0.623	0.598	0.553	0.648
	• 30–39	0.670	0.582	0.771	0.468	0.414	0.529	0.539	0.491	0.592
	• 40–49	0.519	0.440	0.612	0.570	0.497	0.653	0.552	0.497	0.613
	• 50–59	0.534	0.451	0.632	0.562	0.470	0.672	0.541	0.479	0.611
	• 60+	0.546	0.483	0.617	0.678	0.520	0.884	0.551	0.495	0.613
	• Pandemic (vs prepandemic)	1.055	0.963	1.157	0.673	0.618	0.733	0.830	0.779	0.885
	• Site (Kibera vs Asembo)							2.298	2.152	2.455
SARI	• Male (vs female)	0.943	0.858	1.037	0.887	0.813	0.968	0.914	0.857	0.974
	Age category (ref < 5 years)									
	• 5–17	0.830	0.744	0.926	1.048	0.943	1.164	0.931	0.864	1.004
	• 18–29	0.844	0.703	1.014	0.966	0.837	1.115	0.919	0.822	1.027
	• 30–39	0.919	0.741	1.140	0.748	0.631	0.887	0.808	0.705	0.925
	• 40–49	0.843	0.669	1.061	0.830	0.691	0.996	0.833	0.721	0.961
	• 50–59	0.712	0.556	0.913	0.691	0.536	0.890	0.701	0.587	0.837
	• 60+	0.677	0.563	0.816	0.840	0.567	1.245	0.712	0.604	0.838

AFI, Acute febrile illness; ARI, Acute Respiratory Illness; SARI, Severe Acute Respiratory Illness; aOR, adjusted odds ratio; CI, confidence interval.

The site-specific adjusted effect estimates of reported healthcare seeking at a chemist with any illness or select clinical syndromes in the pandemic compared to the prepandemic period are shown in Table 6. We observed higher odds of reported healthcare seeking at chemists during the pandemic relative to the prepandemic period for any illness in Asembo (aOR: 1.23; 95% CI: 1.20–1.27), and in Kibera (aOR: 1.40; 95% CI: 1.35–1.46). A similar pattern was observed for the odds of reported healthcare seeking at the chemists with AFI, ARI, and ARI in both Asembo and Kibera (aORs > 1). For the combined analysis, the odds of Kibera participants reporting healthcare seeking at a chemist with any illness was lower than their counterparts in Asembo (aOR: 0.80; 95% CI: 0.78–0.83). Male and participants aged 5–59 years were less likely to report seeking healthcare at health facilities with any illness (aORs <1, Tables 2–5) but more likely to report seeking healthcare at a chemist (aORs>1, Table 6) than their counterparts.

**Table 6:** Effect of COVID-19 pandemic on healthcare seeking at the chemists with any illness and select clinical syndromes adjusted for age and gender in Asembo, Siaya County and Kibera, Nairobi County, Kenya.

Syndromes		Asembo			Kibera			Both sites		
	Characteristic variables	aOR	95% CI		aOR	95% CI		aOR	95% CI	
Any illness	• Pandemic (vs prepandemic)	1.231	1.198	1.265	1.402	1.347	1.461	1.283	1.254	1.313
	• Site (Kibera vs Asembo)							0.804	0.783	0.827
	• Male (vs female)	1.056	1.024	1.089	1.190	1.137	1.244	1.099	1.072	1.127
	Age category (ref < 5 years)									
	• 5–17	1.168	1.120	1.217	1.129	1.063	1.199	1.159	1.120	1.200
	• 18–29	1.079	1.022	1.139	1.218	1.139	1.302	1.131	1.084	1.180
	• 30–39	1.209	1.138	1.284	1.384	1.285	1.490	1.277	1.218	1.338
	• 40–49	1.254	1.174	1.339	1.368	1.260	1.486	1.300	1.235	1.369
	• 50–59	1.103	1.032	1.178	1.155	1.038	1.284	1.131	1.069	1.196
	• 60+	0.865	0.821	0.912	0.926	0.790	1.084	0.883	0.841	0.926
	• Pandemic (vs prepandemic)	1.393	1.318	1.473	1.331	1.180	1.501	1.381	1.313	1.453
	• Site (Kibera vs Asembo)							0.768	0.718	0.821
AFI	• Male (vs female)	1.116	1.053	1.183	1.251	1.109	1.411	1.141	1.082	1.210
	Age category (ref < 5 years)									
	• 5–17	1.356	1.261	1.458	1.179	1.001	1.389	1.321	1.237	1.412
	• 18–29	1.110	0.997	1.236	1.061	0.880	1.279	1.103	1.005	1.210
	• 30–39	1.370	1.204	1.558	1.293	1.062	1.574	1.359	1.221	1.513
	• 40–49	1.426	1.232	1.651	1.080	0.861	1.354	1.315	1.164	1.486
	• 50–59	1.110	0.956	1.290	1.158	0.879	1.525	1.134	0.994	1.293
	• 60+	1.129	0.997	1.278	0.523	0.324	0.844	1.070	0.951	1.205
	• Pandemic (vs prepandemic)	1.287	1.235	1.342	1.446	1.369	1.528	1.344	1.300	1.389
ARI	• Site (Kibera vs Asembo)							0.766	0.738	0.795
	• Male (vs female)	1.058	1.012	1.107	1.187	1.119	1.259	1.101	1.063	1.142
	Age category (ref < 5 years)									
	• 5–17	1.021	0.962	1.083	1.160	1.075	1.252	1.074	1.025	1.125
	• 18–29	1.017	0.939	1.101	1.179	1.079	1.288	1.082	1.020	1.148
	• 30–39	1.168	1.068	1.276	1.375	1.246	1.517	1.254	1.174	1.339
	• 40–49	1.230	1.116	1.355	1.352	1.210	1.511	1.282	1.192	1.379
	• 50–59	1.147	1.040	1.265	1.131	0.978	1.309	1.165	1.074	1.263
	• 60+	0.823	0.763	0.887	0.948	0.754	1.191	0.861	0.804	0.923
	• Pandemic (vs prepandemic)	1.455	1.354	1.564	1.657	1.518	1.809	1.537	1.454	1.625
SARI	• Site (Kibera vs Asembo)							0.681	0.642	0.722
	• Male (vs female)	1.100	1.022	1.184	1.129	1.030	1.237	1.110	1.049	1.176
	Age category (ref < 5 years)									
	• 5–17	1.147	1.053	1.250	1.121	1.002	1.255	1.132	1.058	1.211
	• 18–29	1.184	1.028	1.365	1.078	0.925	1.256	1.132	1.021	1.256
	• 30–39	1.471	1.235	1.751	1.224	1.030	1.454	1.338	1.185	1.511
	• 40–49	1.482	1.242	1.768	1.166	0.972	1.400	1.313	1.158	1.489
	• 50–59	1.336	1.111	1.607	1.283	1.005	1.639	1.319	1.139	1.527
	• 60+	1.052	0.918	1.206	1.022	0.672	1.554	1.037	0.914	1.177

AFI, Acute febrile illness; ARI, Acute Respiratory Illness; SARI, Severe Acute Respiratory Illness; aOR, adjusted odds ratio; CI, confidence interval.

## Discussion

We utilized a well-characterized longitudinal population-based infectious disease surveillance platform in a rural setting and an urban informal settlement in Kenya to investigate the impact of the COVID-19 pandemic on reported healthcare seeking at health facilities and at chemists among participants who reported illnesses over a seven-year period, January 2016 to August 2022. We observed a 19% decline in the odds of reported healthcare seeking at a health facility with any illness during the pandemic in Asembo (rural setting) and 30% decline in Kibera (urban informal settlement). Despite provision of free medical care at the surveillance health facilities in both sites, COVID-19 pandemic was associated with a 5% decline in the odds of reported healthcare seeking with any illness in Asembo and a 26% decline in Kibera. The decline in reported healthcare seeking at surveillance health facilities was also observed for the other clinical syndromes in both sites except for AFI in Asembo that had a 18% increase in healthcare seeking. The pandemic was associated with increased healthcare seeking at chemists in both sites among participants who reported any illness and those with AFI, ARI, and SARI. Participants in Kibera were more likely to seek healthcare at a health facility compared to participants in Asembo.

The observed reduction in healthcare seeking at health facilities and increased utilization of chemists in the two surveillance sites during the COVID-19 pandemic could be partly attributed to the following reasons. First, the pandemic likely instilled fear among the participants of possible exposure to SARS-CoV-2 infections at the health facilities, resulting in people opting for treatment from alternative sources, such as a chemist, as shown in this study, and traditional healers/medicine or self-treatment at home as reported in the literature [8]. Second, in the initial phase of the pandemic response, the Kenyan government introduced isolation in COVID-19 centers for those infected with SARS-CoV-2 and quarantine of contacts to mitigate further spread of the virus. This might have discouraged those with mild infections from seeking healthcare outside of their homes [20]. With the exponential increase in infection, isolation was replaced by home-based care of mild COVID-19 cases to reduce the burden on healthcare facilities. Third, hospitals in Kenya were hesitant to admit patients with ‘red flag’ symptoms of COVID-19 and some were partially closed or closed in the early phase of the pandemic for fear of in-hospital transmission of infections and limited routine services. Fourth, the fear of COVID-19 testing that involved an uncomfortable nasopharyngeal (NP) swab collection might have influenced the utilization of health facilities, especially in facilities where SARS-CoV-2 surveillance was routine. Fifth, travel restrictions, such cessation of movements in areas that had high infection rates and imposed curfews, might have discouraged patients from seeking healthcare. Finally, high treatment costs for COVID-19 and related complications hindered patients from seeking healthcare, especially at referral health facilities not covered by PBIDS. Noteworthy, the substantial reduction in healthcare seeking in the two surveillance sites are in line with findings from other studies: there are documented reports of patients refraining from medical appointments [6], deferring clinic visits [7], patients with COVID-19-like symptoms not seeking formal medical care [11], reduction in inpatient/outpatient healthcare utilization [12,21–23], and reduced psychological support and maintenance of health [5] during COVID-19 pandemic. The observed increase in healthcare seeking at the surveillance health facility in Asembo during the pandemic might have been driven by increased malaria circulation given that Asembo is malaria-endemic [17]. Similar to the findings in this study, a Swiss study among adults found an increased utilization of pharmacies [1]. The pharmacies were easily accessible and offered low-threshold health services [1]. The higher likelihood of healthcare seeking at Tabitha Medical Clinic than at St. Elizabeth Lwak Mission Hospital might be attributable to the proximity (distance) to the surveillance health facility. Asembo covers a wider catchment area, that is within a five-kilometer radius of St. Elizabeth Lwak Mission Hospital, compared with Kibera that covers a one-kilometer radius around Tabitha Medical Clinic [15]. The effect of distance on health care utilization in PBIDS is documented [24]. The observed overall decline in healthcare seeking in the two surveillance areas might result in increased transmission and health burden of prevalent diseases in the community such as HIV. In contrast, the observed increased healthcare seeking at chemist might result in misuse of unprescribed drugs and increased risk of drug resistance in the community. Thus, the findings from this study could be crucial to public health practitioners and policy makers in developing health promotion strategies targeting the at-risk-groups in case of future pandemics.

This study had a few limitations. First, the reported healthcare seeking data were collected every six months and later every four months from September 2021. During a household visit, a participant was asked whether they sought care in the past two weeks from the date of the interview. Because of this shorter queried healthcare seeking period (past two weeks) relative to the between-household visit interval (4 or 6 months), there might be underreporting, especially for healthcare sought proximal to the preceding household visit. Second, the self-reported household morbidity data are prone to recall bias, which might bias effect estimates–though the short period for recall was designed to mitigate the recall bias. Third, household visit was temporarily stopped between April to September 2020 to mitigate the spread of COVID-19 infection and we might have missed crucial information on healthcare utilization in that period. Fourth, the estimated effects of the pandemic on healthcare seeking in this study cohort might have been biased by other factors/unmeasured confounding, such as the economic impact of COVID-19, that were not adjusted for in the regression analyses. Finally, we derived our findings from two unique populations–a rural setting and an urban informal settlement. These two populations are not representative of the Kenyan population and our findings may not be generalizable, especially since healthcare is free for the PBIDS participants.

This study underscores the interruption to healthcare utilization in a resource-limited setting occasioned by COVID-19 pandemic. The pandemic resulted in decline in healthcare seeking at health facilities but a rise in utilization of healthcare at chemists among PBIDS participants with illness.
